# Supplementary material for: STK11 (LKB1) missense somatic mutant isoforms promote tumor growth, motility and inflammation
Source: Commun Biol. 2020 Jul 9;3:366. doi: 10.1038/s42003-020-1092-0 (PMC7347935; doi:10.1038/s42003-020-1092-0)

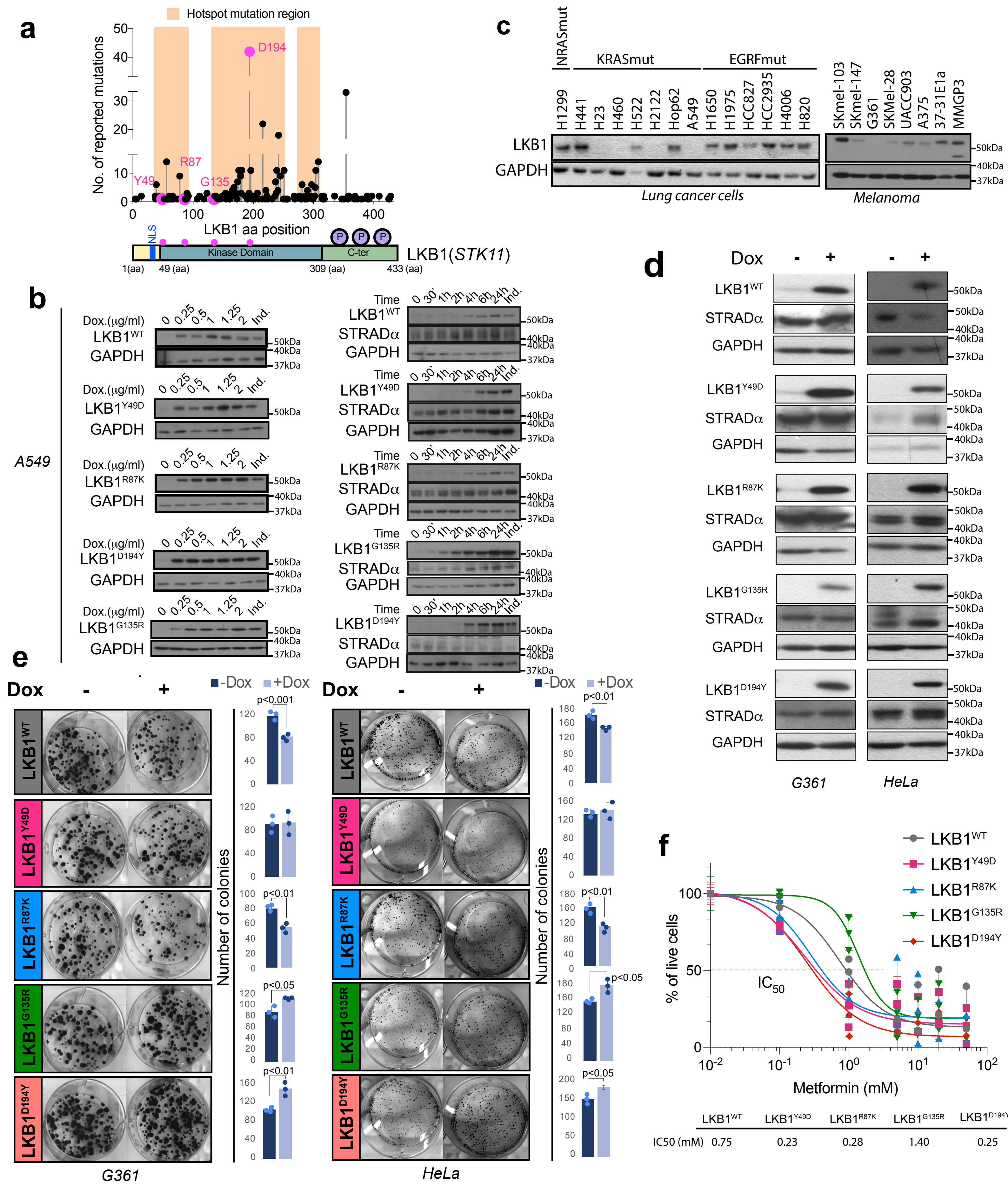

Supplementary Figure 1: LKB1^{Y49D}, LKB1^{G135R} and LKB1^{D194Y} mutants lack the LKB1^{WT} tumor suppressor activity in melanoma and cervix cancer cells.(**a**) Graph and scheme showing the number of STK11 reported mutations (all TCGA studies) and their relative position in the primary structure of LKB1. PInk dots highlight the mutations investigated in this study. (**b**) Titration of Doxycyclin for LKB1 induction. Left panels, cells were treated for the indicated concentration of doxycyclin for 24h. Western-blot shows the amounts of LKB1. GAPDH is showed as loading control. Right panels, cells were treated with 1μg/ml Doxycyclin forn tthe indicated times. Ind.=stable induced isoform for at least 5 days.Western-blot shows the amounts of LKB1 and STRADα. GAPDH is showed as loading control. (**c**) Western-blot showing the amounts of endogenous LKB1 protein in several lung cancer and melanoma cell lines. GAPDH is showed as loading control. (**d**) Wester-blot showing the induction (1μg/ml Doxycyclin) of LKB1 isoforms expression and STRADα in infecteted Hela and G361 cells.GAPDH is showed as loading control. (**e**) Colony formation assay of Hela and G361 cells infected with constructs containing the different LKB1 mutatns isoforms. Graphs show the number of colonies. Representative images are showed. All experiments were done in triplicates. (**f**) Viability assay of A549 cells expressing the different LKB1 isoformsexposed to increasing concentrations of metformin for 48h (n=3, *p*-value was calcutlated by student *t*-test). Table shows the cal-culated IC50 for cell lines expressing each isoform.

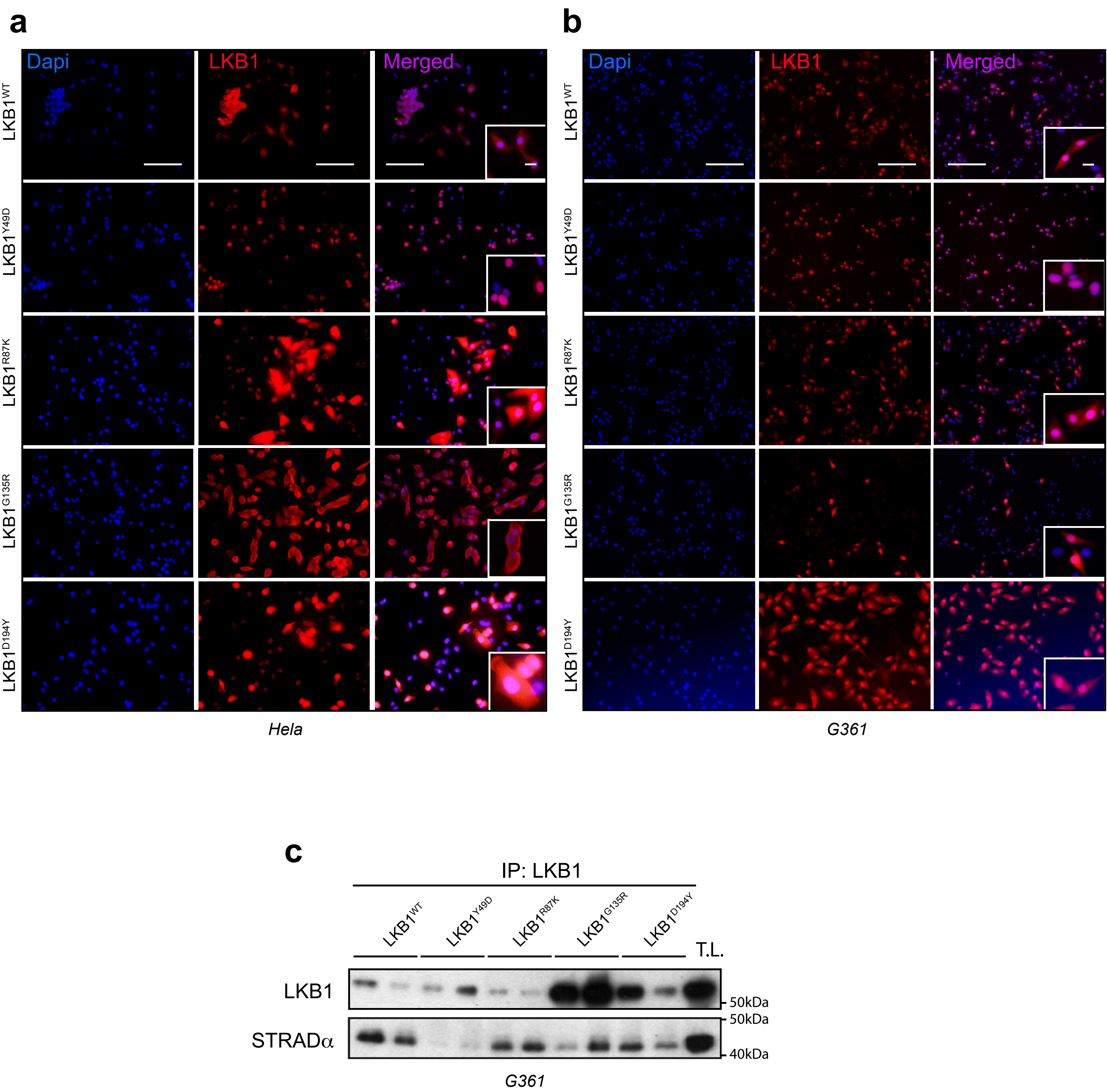

Supplementary Figure 2: Subcellular localization of LKB1 mutant isoforms and binding capabilities to STRADα. (**a**) Immunofluoresce showing the LKB1 localization in Hela cells expressing the different LKB1 mutant isoforms. Dapi shows nuclear staining. Insets show magnification of cells expressing LKB1 isoform. Bars represent 500μm and 100μm. (**b**) Same as in (A) using G361 cells. (**c**) Western blot showing the the amount of STRADα bound to the different LKB1 mutatn isoforms in G361 cells as in Figure 2C.Samples are showed in duplicates.Total lysate (50μg protein) (T.L.) is used as a control for enrichment.

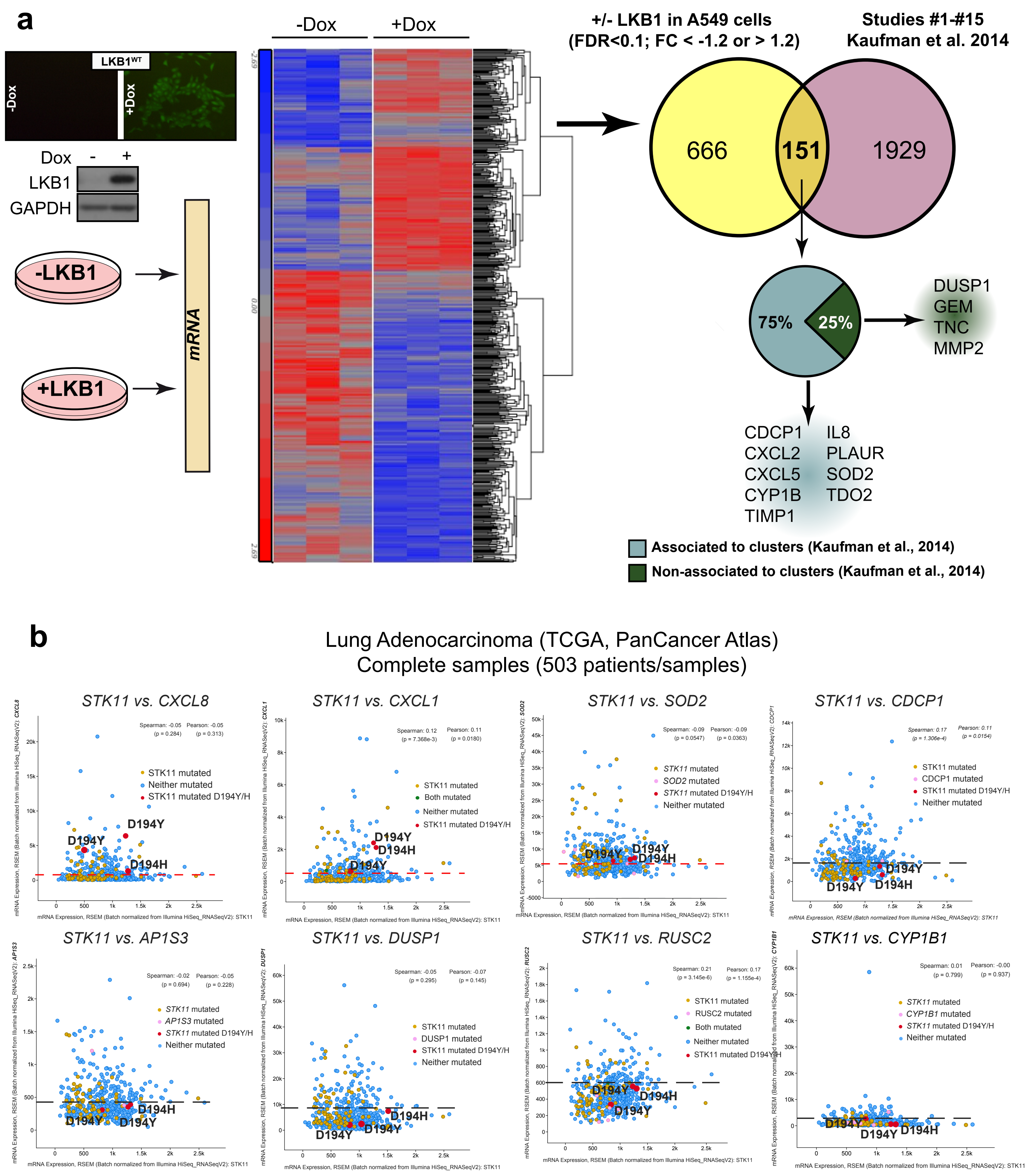

Supplementary Figure 3: Expression of LKB1 mutant isoforms promote gene expression regulation (a) Expression of LKB1^{WT} isoform in A549 cells was induced upon doxyxyclin treatment(1µg/ml). Gene expression analysis of cells expressing or not expressing LKB1^{WT} was performed (*n*=3 per condition).heatmap shows the unsupervised hierarchical clustering of genes showing differential expre-ssion (FDR<0.1) is showed on the right (3 samples per condition). On the right Venn diagram showing common genes between this study (FDR<0.1; FC < -1.2 or > 1.2) and the unique regulated genes from 15 different datasets (top 200 in each study) studying gene expression profiles associated to LKB1 loss (Kaufman et al.,2014). (**b**) Graphs showing the correlation between the expression of STK11 and the regulated genes by LKB1^{D194Y} in Figure 4d in the lung adenocarcinoma (TCGA, PanCancer Atlas) dataset. Red and black lines shows the lowerand higher expression amount for the gene investigated in the D194 residue mutated samples, respectively.

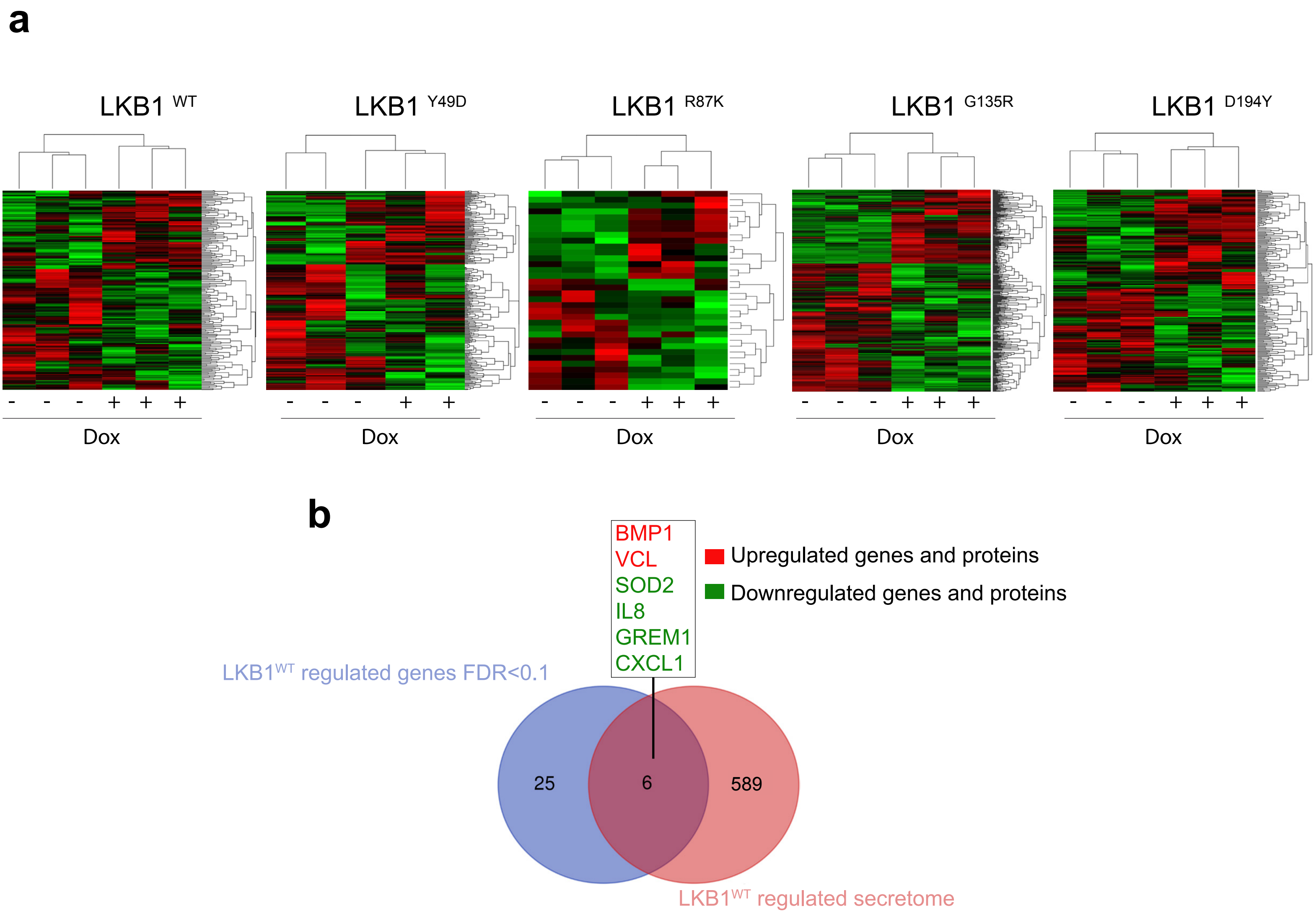

Supplementary Figure 4: Expression of LKB1 mutant isoforms promote secretome regulation: (**a**) Heat-maps of the unsupervised hierarchical clustering of secretome sample based on label free abundance. The entire dataset of database matches for each secretome was used to direct a hierarchical clustering analysis, with log transformed label free quantification being used as the parameter. Samples from un-induced (-) or induced (+) LKB1 isoform are indicated (n=3 per condition, except for Y49D induced n=2). (**b**) Venn diagram comparing the 31 top transcriptionally regulated genes and the secretome identified proteins upon $LKB1^{WT}$ expression.

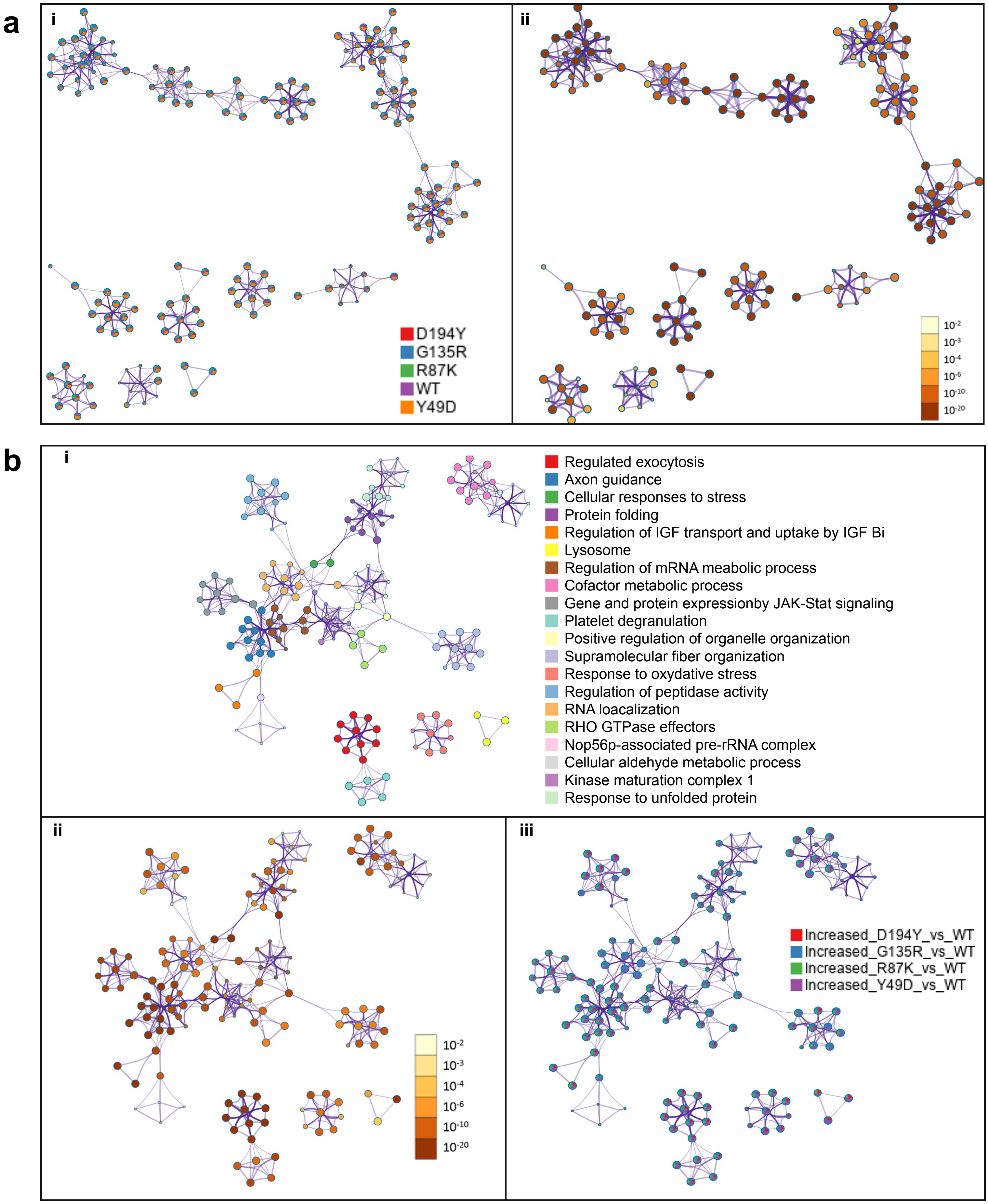

Supplementary Figure 5: **Pathway, process and protein-protein enrichment analysis.** (**a**) Network of enriched terms after LKB1 expression isoforms showed in Figure 4A **(A)** Network of enriched terms represented as pie charts, where pies are color-coded based on the identities of the gene lists (i). (ii) Colored by *p*-value, where terms containing more genes tend to have a more significant *p*-value. **(b)** Network of enriched terms upregulated in mutant LKB1 isoforms compared to wild type: (i) colored by cluster ID, where nodes that share the same cluster ID are typically close to each other; (ii) colored by *p*-value, where terms containing more genes tend to have a more significant *p*-value. (iii) Network of enriched terms represented as pie charts, where pies are color-coded based on the identities of the gene lists.

Originals Figure 1c

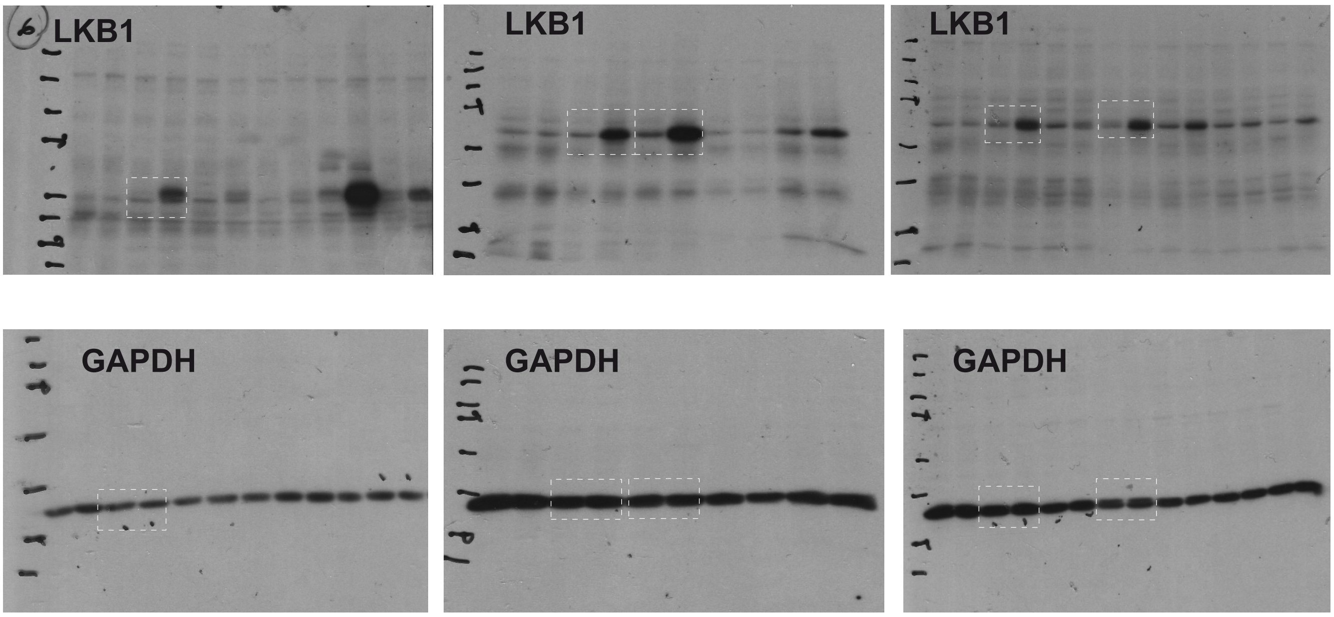

Original data Figure 2c

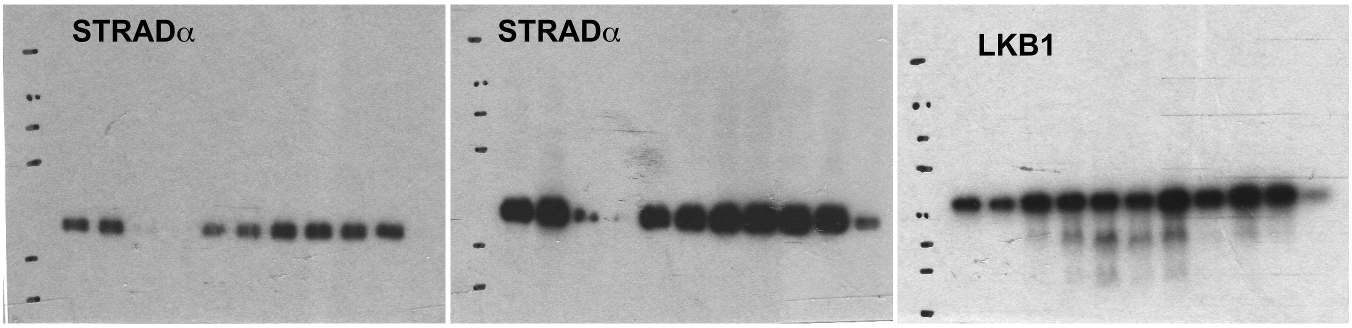

Original data Figure 2d

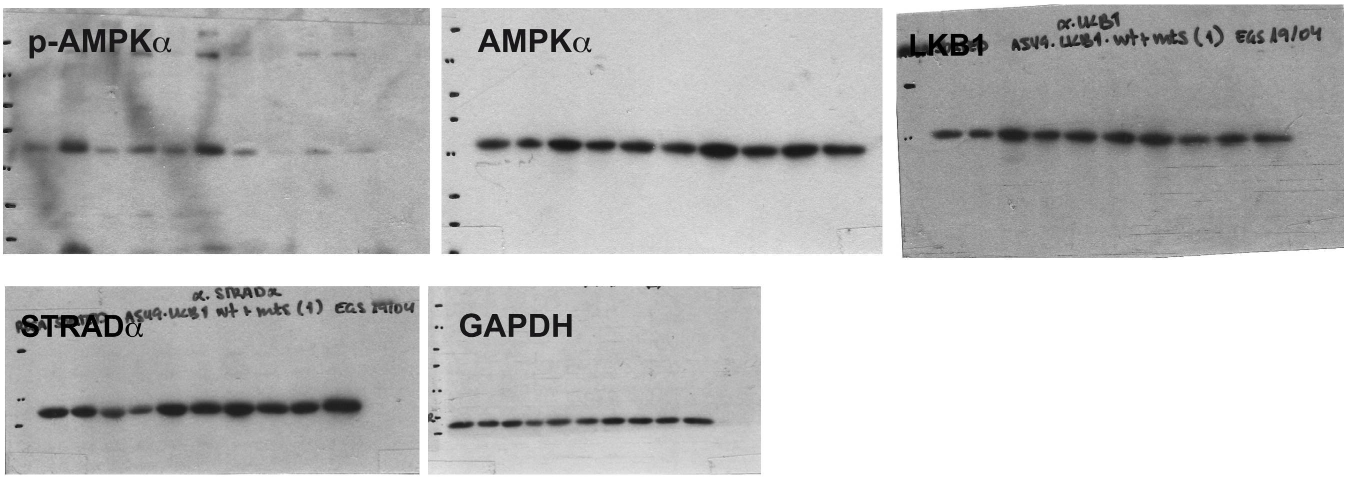

Original data Figure 2e

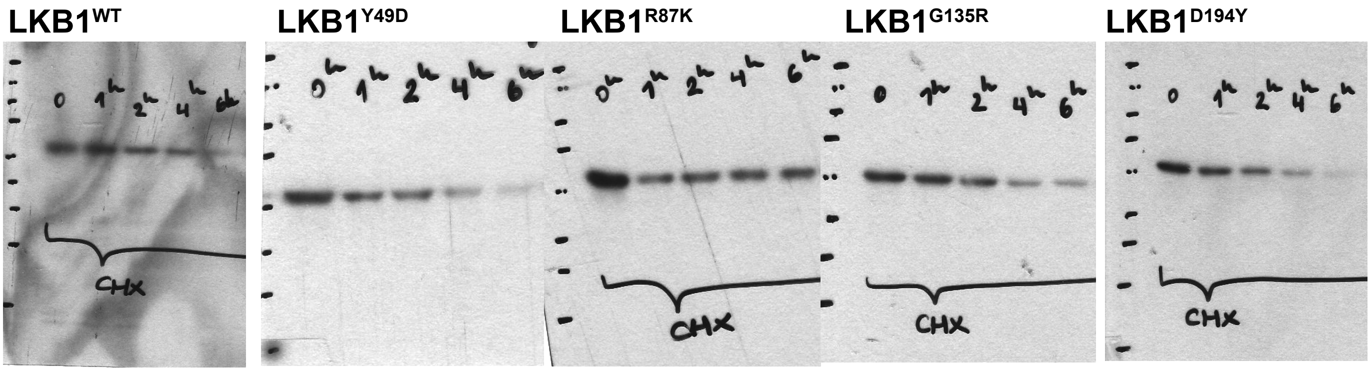

Original data Figure 3a

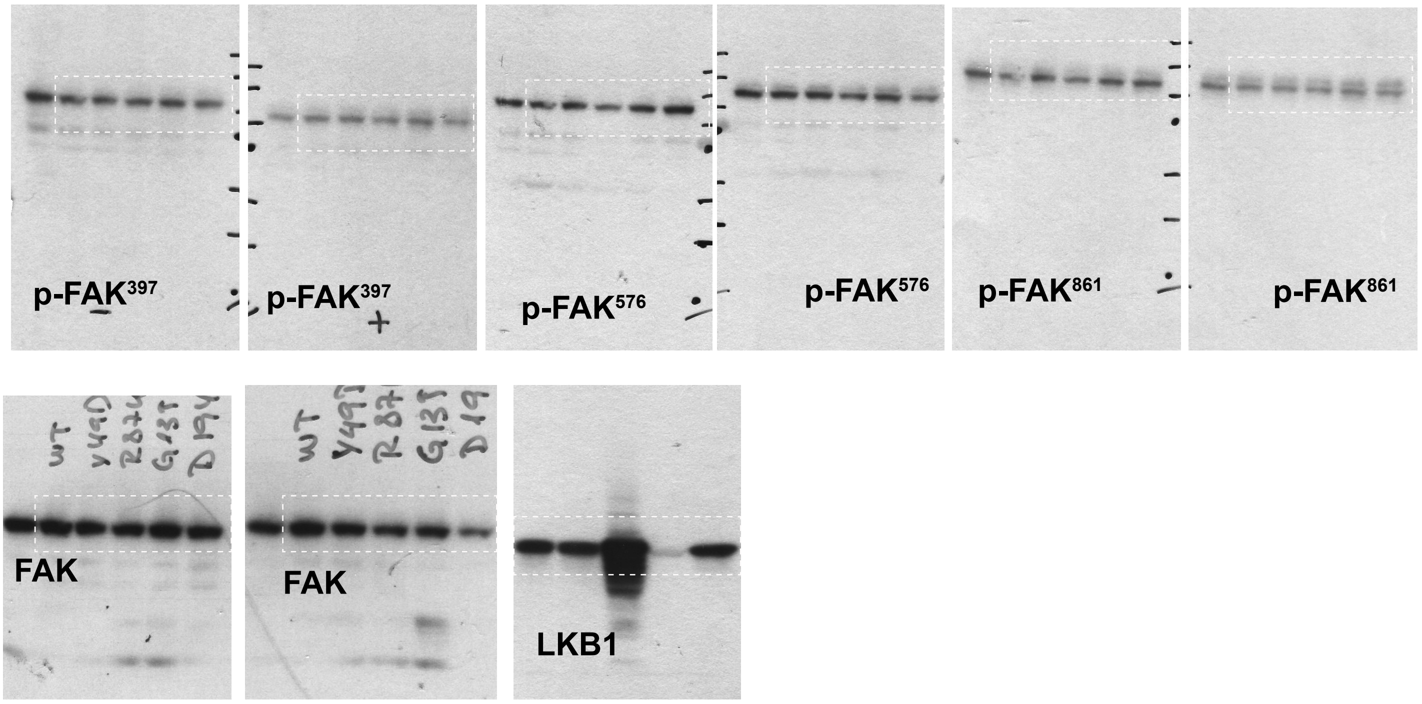

Original data Figure 3e

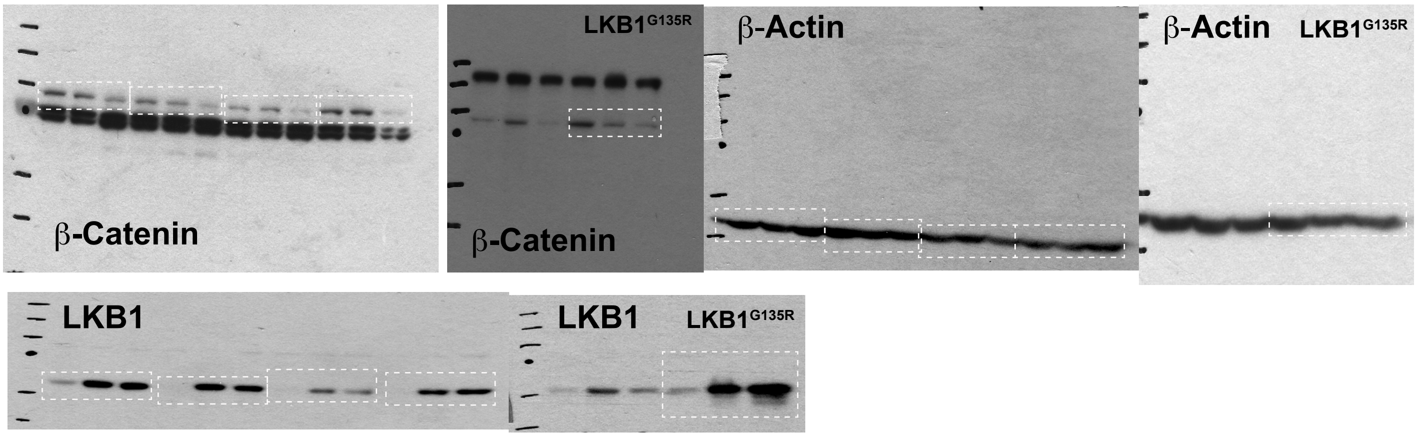

Original data Figure 5c

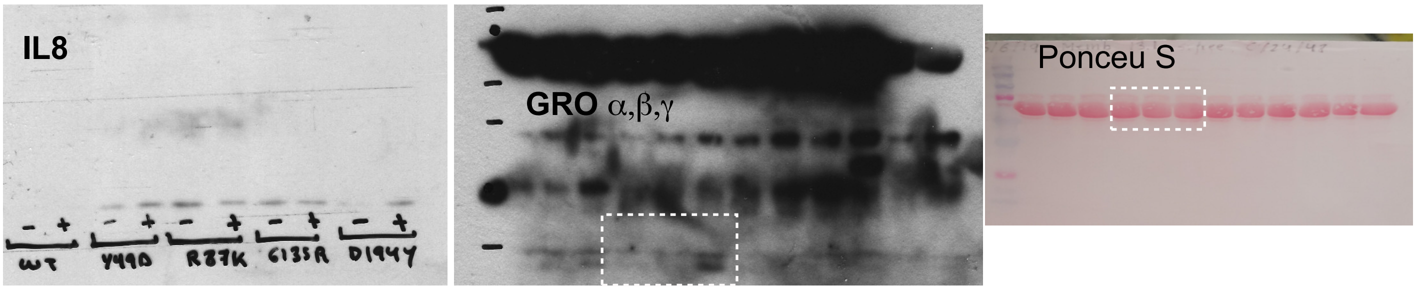

Original data Figure 5d

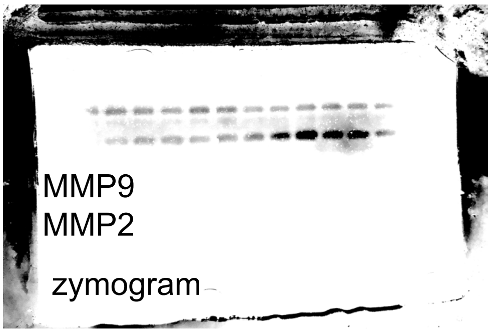

Original data Supplementary Figure 1b

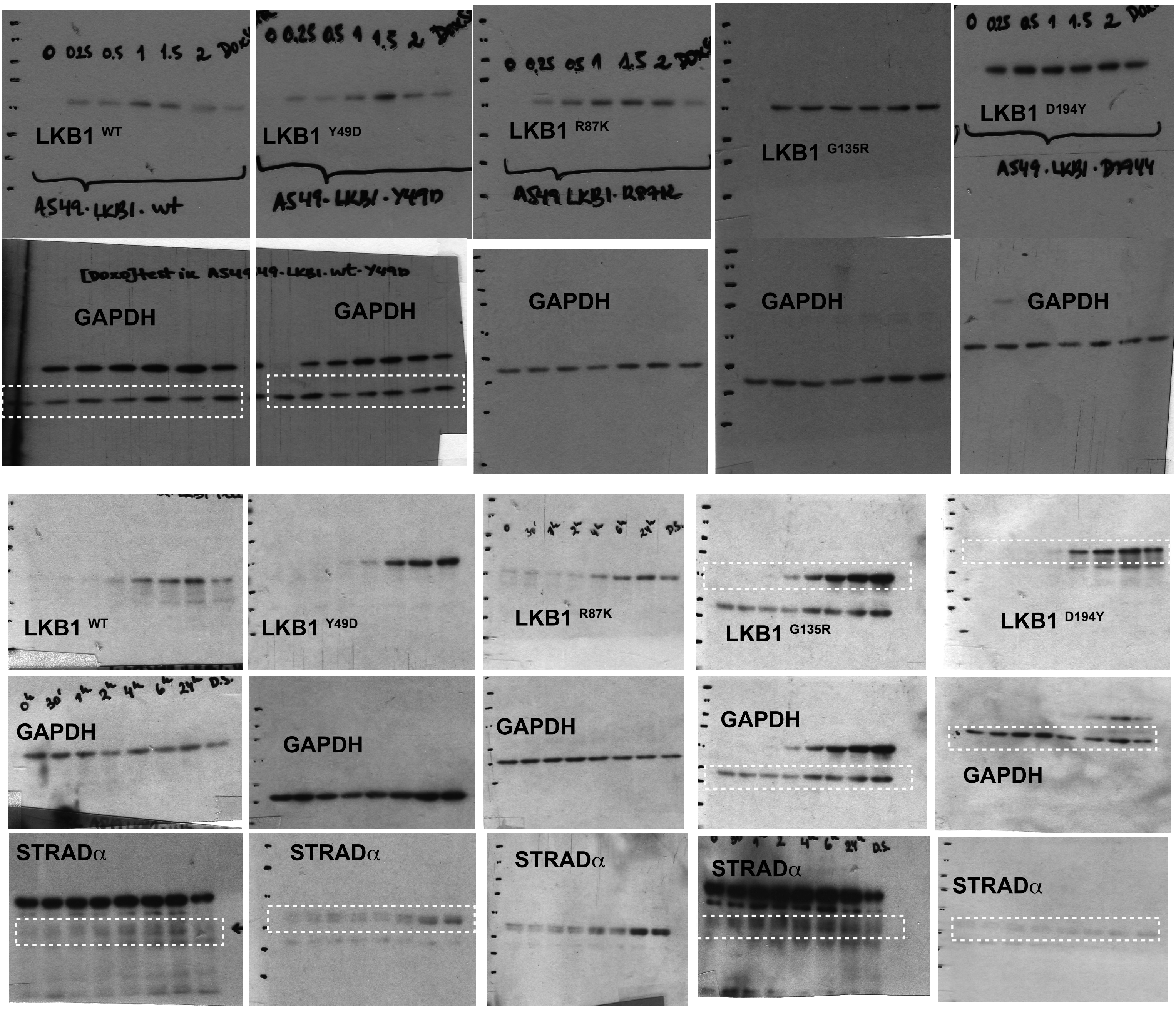

Original data Supplementary Figure 1c

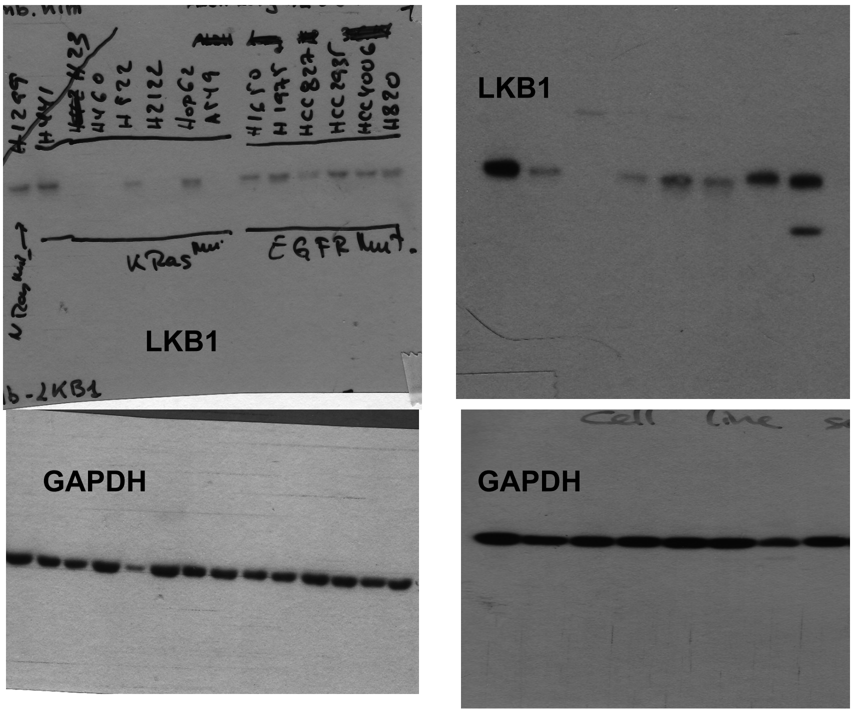

Original data Supplementary Figure 1c

G361 CELLS

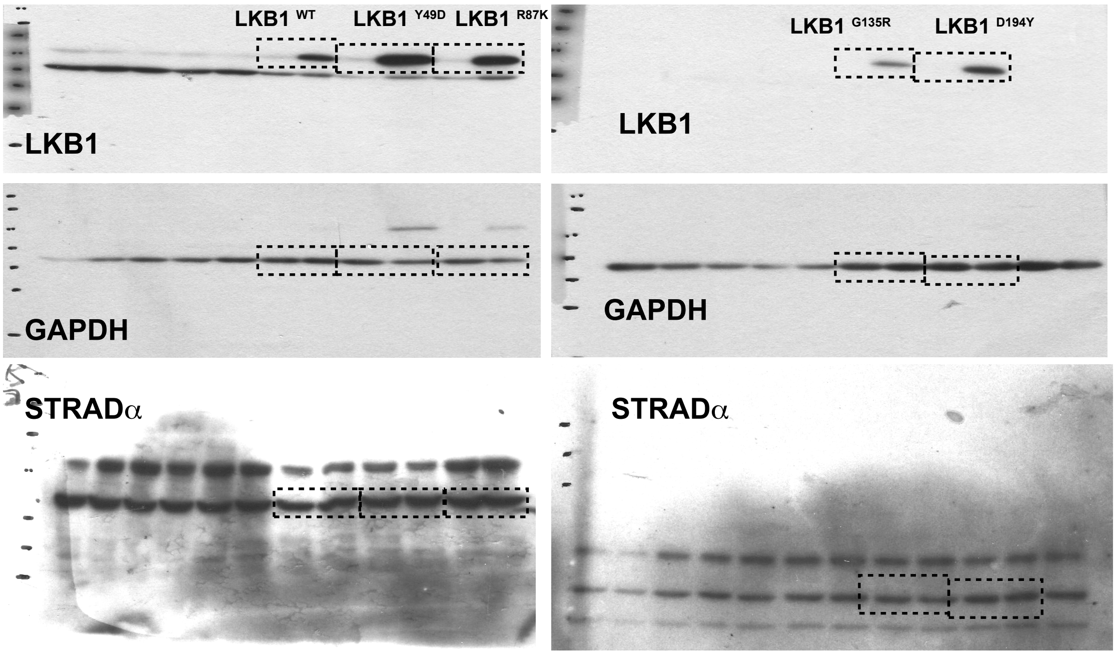

HeLa CELLS

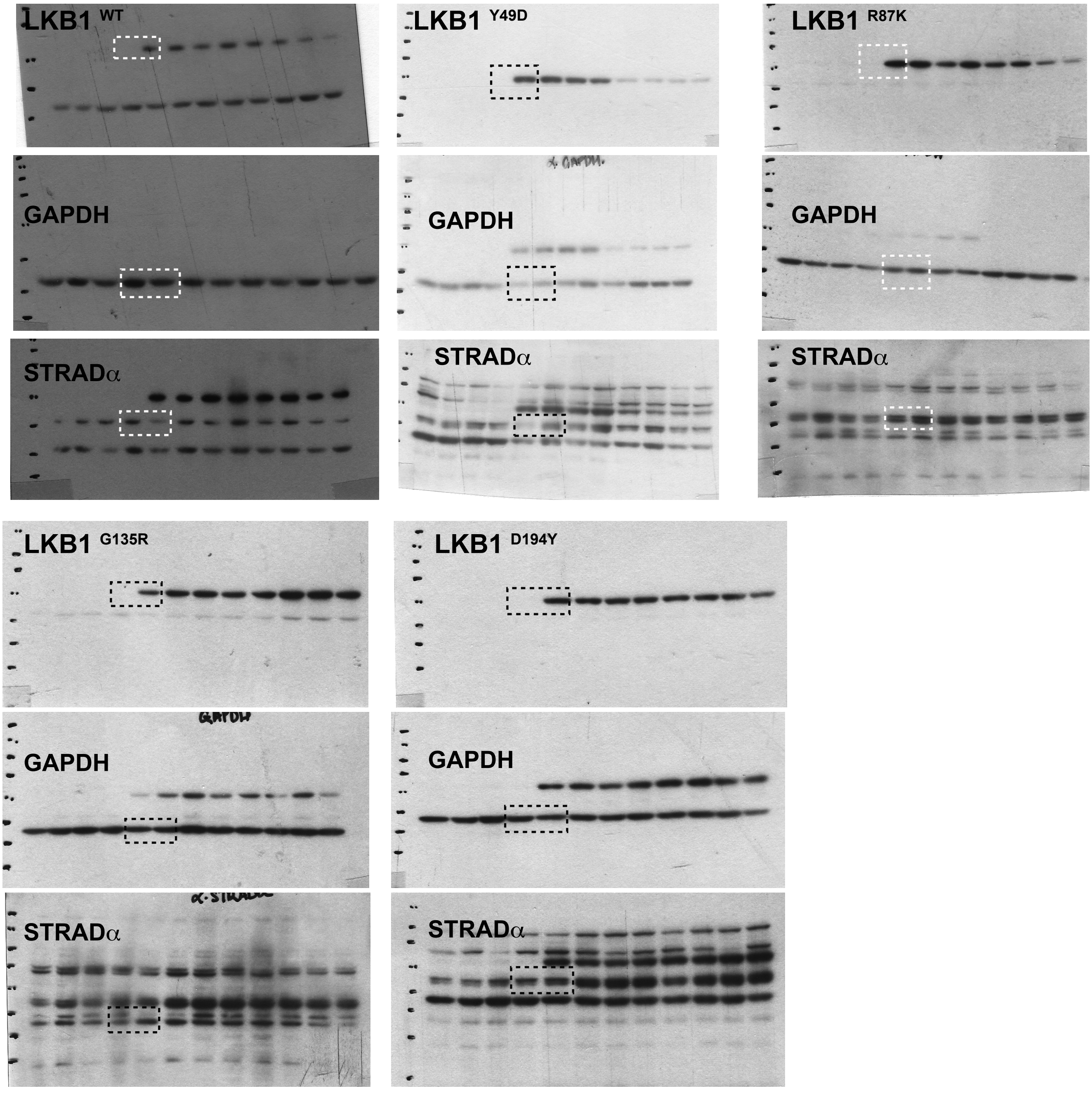

Original data Supplementary Figure2c

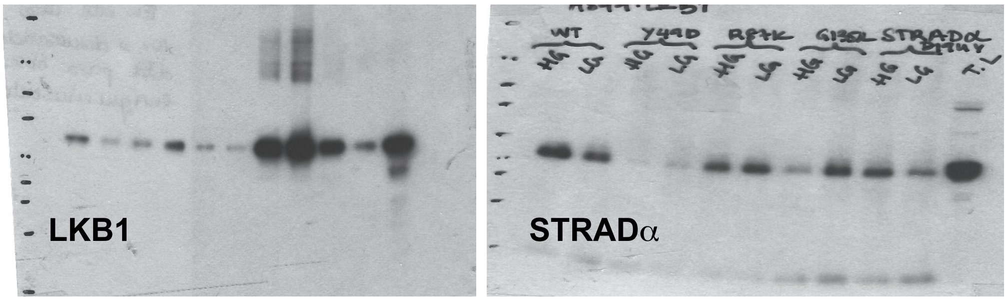

Supplement: Supplementary file 1 — Supplementary Information [file 42003_2020_1092_MOESM1_ESM.pdf]
