# Supplementary material for: STK11 (LKB1) missense somatic mutant isoforms promote tumor growth, motility and inflammation
Source: Commun Biol. 2020 Jul 9;3:366. doi: 10.1038/s42003-020-1092-0 (PMC7347935; doi:10.1038/s42003-020-1092-0)
Supplement: Supplementary file 2 — Description of Additional Supplementary Files [file 42003_2020_1092_MOESM2_ESM.pdf]

Descriptions of Additional Supplementary Files

Supplementary data 1: Proteins detected by mass spectrometry in secretomes before and after the expression of the different LKB1 isoforms. Samples are showed in triplicates.

Supplementary data 2: Proteins differentially detected by mass spectrometry in secretomes after the expression of the different LKB1 isoforms.

Supplementary data 3: List of the common proteins identified in the secretomes obtained from cells expressing the different LKB1 isoforms (Fig. 5A)
