# Supplementary material for: STK11 (LKB1) missense somatic mutant isoforms promote tumor growth, motility and inflammation
Source: Commun Biol. 2020 Jul 9;3:366. doi: 10.1038/s42003-020-1092-0 (PMC7347935; doi:10.1038/s42003-020-1092-0)
Supplement: Supplementary file 6 — Reporting Summary [file 42003_2020_1092_MOESM6_ESM.pdf]

nature research

Corresponding author(s): Juan Angel Recio Conde

Last updated by author(s): Nov 11, 2019

Reporting Summary

Nature Research wishes to improve the reproducibility of the work that we publish. This form provides structure for consistency and transparency in reporting. For further information on Nature Research policies, see Authors & Referees and the Editorial Policy Checklist.

Statistics

For all statistical analyses, confirm that the following items are present in the figure legend, table legend, main text, or Methods section.

n/a	Confirmed	
☐	☒	The exact sample size (*n*) for each experimental group/condition, given as a discrete number and unit of measurement
☐	☒	A statement on whether measurements were taken from distinct samples or whether the same sample was measured repeatedly
☐	☒	The statistical test(s) used AND whether they are one- or two-sided<br>*Only common tests should be described solely by name; describe more complex techniques in the Methods section.*
☒	☐	A description of all covariates tested
☒	☐	A description of any assumptions or corrections, such as tests of normality and adjustment for multiple comparisons
☒	☐	A full description of the statistical parameters including central tendency (e.g. means) or other basic estimates (e.g. regression coefficient) AND variation (e.g. standard deviation) or associated estimates of uncertainty (e.g. confidence intervals)
☐	☒	For null hypothesis testing, the test statistic (e.g. *F*, *t*, *r*) with confidence intervals, effect sizes, degrees of freedom and *P* value noted<br>*Give P values as exact values whenever suitable.*
☒	☐	For Bayesian analysis, information on the choice of priors and Markov chain Monte Carlo settings
☐	☒	For hierarchical and complex designs, identification of the appropriate level for tests and full reporting of outcomes
☒	☐	Estimates of effect sizes (e.g. Cohen's *d*, Pearson's *r*), indicating how they were calculated

Our web collection on statistics for biologists contains articles on many of the points above.

Software and code

Policy information about availability of computer code

Data collection	IVIS Spectrum In Vivo Imaging System, MASCOT,
Data analysis	ImageJ, Prism 6.0, Scaffold 4.0, UCSF Chimera, Cytoscape 3.7.1

For manuscripts utilizing custom algorithms or software that are central to the research but not yet described in published literature, software must be made available to editors/reviewers. We strongly encourage code deposition in a community repository (e.g. GitHub). See the Nature Research guidelines for submitting code & software for further information.

Data

Policy information about availability of data

All manuscripts must include a data availability statement. This statement should provide the following information, where applicable:

- Accession codes, unique identifiers, or web links for publicly available datasets
- A list of figures that have associated raw data
- A description of any restrictions on data availability

The Arrayexpress accession number is E-MTAB-8863. Mass spectometry accession number PXD018041

Field-specific reporting

Please select the one below that is the best fit for your research. If you are not sure, read the appropriate sections before making your selection.

☒ Life sciences ☐ Behavioural & social sciences ☐ Ecological, evolutionary & environmental sciences

For a reference copy of the document with all sections, see nature.com/documents/nr-reporting-summary-flat.pdf

Life sciences study design

All studies must disclose on these points even when the disclosure is negative.

Sample size	No statistical methods were used to determine sample size
Data exclusions	No data was excluded from the analysis.
Replication	Figure 1d: Data are representative of three independent experiments. Quantifications show the mean and SD of the three experiments<br>Figure 1e, f Data are representative of three independent experiments Data show the mean and the SD,<br>Figure 1g Data was performed in cuadruplicagtes Data represent mean and their SD<br>Figure 2a: Data represent the mean of four independent experiments and their SD.<br>Figure 2c :Data are representative of two independent experiments.<br>Figure 2d, e: Data are representative of three independent experiments.<br>Figure 3b : Data represent the mean of between 35 and 50 independent cell movement counts per isoform and their SD.<br>Figure 3d: Data are representative of three independent experiments.<br>Figure 3e:Data are representative of three independent experiments.<br>Figure 3f:Data are representative of two independent experiments.<br>Figure 4b: Three biological replicates were used to acquired the data<br>Figure 4c-d: Data represent the mean of three independent experiments and their SD.<br>Figure 5a: Three biological replicates were used to acquired the data<br>Figure 5c-d: Data are representative of two independent experiments.
Randomization	Animals were assigned randomly to experimental and control groups
Blinding	Investigators were not blinded during data collection

Reporting for specific materials, systems and methods

We require information from authors about some types of materials, experimental systems and methods used in many studies. Here, indicate whether each material, system or method listed is relevant to your study. If you are not sure if a list item applies to your research, read the appropriate section before selecting a response.

Materials & experimental systems

n/a	Involved in the study	
☐	☒	Antibodies
☐	☒	Eukaryotic cell lines
☒	☐	Palaeontology
☐	☒	Animals and other organisms
☒	☐	Human research participants
☒	☐	Clinical data

Methods

n/a	Involved in the study	
☒	☐	ChIP-seq
☒	☐	Flow cytometry
☒	☐	MRI-based neuroimaging

Antibodies

Antibodies used

Mouse monoclonal anti-LKB1 (ley37D/G6) (WB) Santa Cruz Biotechnology Cat# sc-32245 RRID:AB_627890

Mouse monoclonal anti-IL8 (B-2) Santa Cruz Biotechnology Cat# sc-8427, RRID:AB_627331

Mouse monoclonal anti-GRO-alpha , beta gamma (A-6)(CXCL1,2,3) Santa Cruz Biotechnology Cat# sc-365870

Rabbit monoclonal anti-LKB1 (D60C5F10) (IHC-P) Cell signaling Cat# 13031 RRID:AB_2716796

Rabbit polyclonal anti-beta-catenin Cell signaling Cat#9562 RRID:AB_331149

Rabbit monoclonal anti-Ki67 (SP6) Abcam Cat# ab16667 RRID:AB_302459

Mouse monoclonal CONFIRM anti-Vimentin (V9) Roche Cat# 790-2917 RRID:AB_2687607

Rabbit monoclonal anti-ERG (EPR3864) Novus Cat# NBP1-40794 RRID:AB_2098414

Goat polyclonal anti E-Cadherin R&D systems Cat# AF648 RRID:AB_355504

Rabbit polyclonal anti-G3PDH/GAPDH Trevigen Cat# 2275-PC-100 RRID: Unknown

Rabbit monoclonal anti LKB1 (27D10) (IP, WB) Cell signaling Cat# 3050, RRID:AB_823559

Mouse monoclonal anti-beta-actin-HRP conjugated clone (AC-15) SIGMA Cat# A3854 RRID:AB_262011

Mouse monoclonal anti-FAK BD Biosciences Cat# 610087, RRID:AB_397494

Mouse monoclonal anti-FAKY397 BD Biosciences Cat# 611723, RRID:AB_399199

Rabbit polyclonal anti-FAKY576 MyBiosource Cat# MBS462199 RRID: Unknown

Rabbit polyclonal anti-FAKY861 Abcam Cat# ab4804, RRID:AB_304641

Goat polyclonal anti-STRADalpha(N-13) Santa Cruz Biotechnology Cat# sc-34102, RRID:AB_669903

Validation

Mouse monoclonal anti-LKB1 (ley37D/G6) (WB)Validated forrecognition of mouse, rat and human origen, for WB, IP, IF y IHC(P)

Mouse monoclonal anti-IL8 (B-2) recognize IL8 of human origen, for WB, IP, IF y ELISA

Mouse monoclonal anti-GROa,b,g (A-6)(CXCL1,2,3) Detects GROα of mouse, rat and human origin, GROβ and GROγ of human origin, and MIP-2 and Cxcl3 of mouse origin and the corresponding rat homologs in WB, IP, IF, IHC(P) y ELISA

Rabbit monoclonal anti-LKB1 (D60C5F10) (IHC-P) Validated for Human, Mouse, Monkey LKB1detection in IHC(P)

Rabbit polyclonal anti-b-catenin Recognize Human, Mouse, Rat, Monkey for WB, IP, IHC

Rabbit monoclonal anti-Ki67 (SP6) Detects Mouse, Rat, Human, Common marmoset for WB and IHC

Mouse monoclonal CONFIRM anti-Vimentin (V9) Detects human Vimentin for IHC(P)

Rabbit monoclonal anti-ERG (EPR3864) Detects human ERG for WB, IF y IHC(P)

Goat polyclonal anti E-Cadherin Detects human and mouse E-Cadherin in direct ELISAs and Western blots.

Rabbit polyclonal anti-G3PDH/GAPDH The antibody detects human and mouse G3PDH for WB

Rabbit monoclonal anti LKB1 (27D10) (IP, WB) Vaidated for Human, Monkey LKB1detection in WB and for IP

Mouse monoclonal anti-b-actin-HRP conjugated clone (AC-15) The antibody cross-reacts with β-Actin expressed in cells of human, bovine, sheep, pig, rabbit, cat, dog, mouse, rat, guinea pig, chicken, carp, and Hirudo medicinalis (leech) tissues,

Mouse monoclonal anti-FAK The antibody detects Human (QC Testing) Mouse, Rat, Dog, Chicken (Tested in Development)

Mouse monoclonal anti-FAKY397 The antibody detects Human (QC Testing)

Rabbit polyclonal anti-FAKY576 The antibody detects Human, Rat and Mouse protein .This antibody recognizes FAK (pY576) with a phosphorylated site at Tyrosine 576. It does not crossreact with non-phosphospecific peptide.

Rabbit polyclonal anti-FAKY861 The antibody detects Human, mouse, and Chicken proteins for WB, IF, IHC-Fr

Goat polyclonal anti-STRADa (N-13) STE20-related kinase adapter protein alpha for WB

Rabbit monoclonal Antibody Anti- AMPK (23A3).RRID:AB_490795

Rabbit polyclonal Antibody Anti- phospho- AMPK 2531, RRID:AB_330330

Eukaryotic cell lines

Policy information about cell lines

Cell line source(s)

lung cancer A549, G361 and Hela cells were purchased form ATCC. A549-luciferase were generated in the lab from A549 purchased cells

Authentication

We assumed the authenticity of cells according to ATCC

Mycoplasma contamination

All cell lines were tested negative for mycoplasma contamination

Commonly misidentified lines
(See ICLAC register)

No cell line listed by ICLAC was used in this study

Animals and other organisms

Policy information about studies involving animals; ARRIVE guidelines recommended for reporting animal research

Laboratory animals	Athymic nu/nu, female 4-6weeks old
Wild animals	The study did not involve wild animals
Field-collected samples	this Study did not involve sample collected from the field
Ethics oversight	Animal experiments were conducted and designed according to protocols approved by the Institutional Animal Care and Use Committee of Vall d´Hebron Institute of Research.

Note that full information on the approval of the study protocol must also be provided in the manuscript.
